# The Cost of Sickness in Texas

**Published:** 1915-03

**Authors:** M. M. Carrick


### The Cost of Sickness in Texas.

BY M. M. CARRICK, M. D.

There is an economic side to the question of sickness that should be considered, as well as that of the inconvenience, loss of time and suffering it entails. The needless and senseless drain upon the public for the care and maintenance of the sick from preventable diseases should cease. It was this aspect of the problem that induced Gladstone, Bismarck, Disraeli and other far-seeing statesmen to incorporate into the laws of their respective governments the statement: "The care of the public health is the first and highest duty of statesmen."

But the lesson comes nearer home. If every public official in Texas would consider the care of the health of the public their first and highest duty; if every physician co-operated with them in the enforcement of sanitary laws, making vaccination against communicable diseases compulsory, for example; if all the people in Texas could and would observe in their daily lives the laws of health, as now known to the scientific world,—sickness would soon decrease and health would abound. Nor is this a chimera. It is a dream that may be realized, for it is a practical, logical solution of our civic problem.

That it is time we awoke to the economic side of the question, if for no other reason, is apparent when we consider that each case of sickness in Texas alone costs $95, on an average, for medical care, drugs, nursing and loss of time, making a yearly tax of about $15,000,000. And this is a conservative estimate, for the actual loss is far beyond these figures.

Professor Irving Fisher, who is now recognized as the world's greatest authority on the conservation of human life, gives us a working basis in dollars and cents. He tells us that the value of a human life is $90 the first years, gradually rising to $4200 when in full vigor. For a period of years it remains stationary, then comes a decline until it becomes negative. He places the average value of lives sacrificed by preventable diseases in the United States at $1700. With these figures as the basis of calculation, and applying them to the 9000 estimated deaths from eight of these preventable diseases last year, we have the sum of $15,300,000. Add to this sum the $15,000,000 which it costs to care for the sick, and we have a total loss of $30,000,000. Statisticians say that although this may seem an enormous estimate, they are underestimated, and that it is entirely possible for the people of

Texas to save more than this every year, if they will. Shall we? The great strides that have already been made in public health should encourage us. We no longer brew disgusting mixtures in gloomy, mysterious laboratories concocted of the skins of vipers, or the powder of spiders, the "eye of the newt and toe of frog," as did the astrologers and magicians of old. We do longer make use of the lancet. Contagious fevers have been controlled and lessened in number as well as fatality; more children arrive at maturity than ever before; we have health boards to care for our cities; quarantine stations to protect the entrance of disease to our shores; but we must still face the fact that many thousands of people die yearly from preventable diseases, and that we pay altogether too little attention to the economic side of this question.

Why not make the care of the public health our first and highest duty, and stop this drain on the civic purse?

---

State Health Department,
Austin, Texas.

Dear Mrs. Daniel: I notice you plan to have life subscribers. I enclose $10 herewith. Put me down first.

Words are inadequate to express my appreciation of the good work of the TEXAS MEDICAL JOURNAL in paving the way and blazing the trail of Progressive Medical Thought in Texas.

All physicians of my age and experience, I believe, will join me in felicitations for your success and the ultimate realization of Dr. Daniel's and your ideals for the profession in Texas.

W. B. COLLINS,
State Health Officer.

Thank you, doctor; such loyalty as yours makes us grateful, indeed.—ED.

